# CHMP5 Is a TCR‐ and Interleukin‐7‐Regulated Protein Required for Naïve T Cell Homeostasis

**DOI:** 10.1002/eji.70310

**Published:** 2026-11-11

**Authors:** Josephine Trichka, Katharine Umphred‐Wilson, Ellen Bak, Yi‐Chu Liao, Jae‐Hyuck Shim, Stanley Adoro

**Affiliations:** ^1^ Experimental Immunology Branch National Cancer Institute National Institutes of Health Bethesda Maryland USA; ^2^ Medical Scientist Training Program Department of Pathology Case Western Reserve University School of Medicine Cleveland Ohio USA; ^3^ Department of Genetic and Cellular Medicine Worcester Massachusetts USA; ^4^ Horae Gene Therapy Center Worcester Massachusetts USA; ^5^ Li Weibo Institute For Rare Diseases Research University of Massachusetts Chan Medical School, Worcester Worcester Massachusetts USA

**Keywords:** CHMP5, homeostasis, interleukin (IL)‐7, naïve T cells, TCR, apoptosis

## Abstract

A diverse repertoire of naïve αβ T cells in the periphery is essential for adaptive immunity to pathogens and cancers, as well as for tissue homeostasis. This peripheral T cell pool is maintained by signals from the T cell receptor (TCR) and interleukin‐7 (IL‐7). However, the mechanisms and downstream factors induced by these signals to support peripheral T cell survival are still not fully understood. We found that TCR and IL‐7 signals posttranscriptionally promoted expression of the charged multivesicular body protein 5 (CHMP5) in naïve CD4^+^ and CD8^+^ T cells, in which CHMP5 functions to prevent oxidative stress and apoptosis. Consequently, inactivation of the *Chmp5* gene in post‐thymic T cells resulted in severe depletion of the peripheral T cell pool by a cell‐intrinsic mechanism. Collectively, our results suggest that induction of CHMP5 by homeostatic TCR and IL‐7 signals is part of a critical mechanism governing naïve T cell homeostasis in the periphery.

## Graphical abstract

TCR and IL‐7 signaling promote CHMP5 expression in mature αβ T cells. Conditional deletion of Chmp5 in post‐thymic T cells is associated with oxidative stress, increased apoptosis, and marked depletion of the peripheral naïve T‐cell pool, supporting a critical role for CHMP5 in mediating peripheral T‐cell homeostasis.

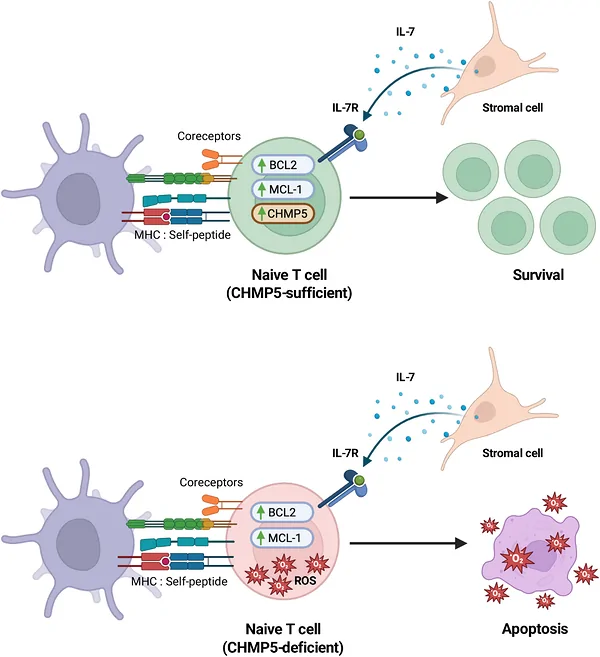

## 1 Introduction

Naïve CD4^+^ and CD8^+^ T cells, which mediate adaptive immunity, are generated in the thymus from CD4^+^CD8^+^ (double positive, DP) thymocytes positively selected by low‐affinity self‐peptide‐major histocompatibility complexes (MHC)‐TCR ligands [1, 2]. Despite daily output from the thymus, the numbers of naïve T cells in secondary lymphoid organs and in the peripheral circulation remain relatively constant in adult mice and humans [3, 4]. Maintenance of this naïve (immunophenotypically defined as CD44^lo^CD62L^hi^CCR7^hi^) T cell pool is regulated by IL‐7 and TCR recognition of self‐peptide‐MHC complexes [5, 6]. Accordingly, IL‐7/IL‐7 receptor (IL‐7R)‐α deficiency [7, 8, 9] or disruption of TCR signals [10, 11, 12] depletes naïve T cells, with the residual T cells in these mice displaying a CD44^hi^ (memory)‐like phenotype and their expansion driven by the increased availability of IL‐7 and self‐peptide‐MHC ligands in these lymphopenic conditions [13]. A major mechanism by which IL‐7 and TCR signals are thought to promote T cell homeostasis is by inducing Bcl‐2 and Mcl‐1, prosurvival factors that inhibit Bim/Bax‐mediated apoptosis [5, 6]. However, inactivation of Bim [14], Bax [15], or overexpression of Bcl‐2 [16, 17] fails to fully restore peripheral naïve T cell numbers under IL‐7‐deficient conditions in vivo, suggesting that the prosurvival effect of IL‐7 is more complex than the simple induction of prosurvival factors. Thus, there remains a need to better understand and elucidate cellular factors induced by homeostatic signals that promote naïve T cell survival.

The charged multivesicular body protein 5 (CHMP5/Vps60/MOS10) was first discovered in yeast to regulate vacuolar protein sorting [18, 19] and was later identified in mammalian cells as an accessory factor for the AAA‐ATPase VPS4 [20, 21, 22] in the endosomal sorting complex required for transport (ESCRT) machinery, which regulates membrane scission events in eukaryotic cells [23, 24]. Extending its function beyond the ESCRT machinery, we uncovered that CHMP5 deficiency in immature DP thymocytes abrogated αβ T cell generation without perturbing ESCRT‐dependent processes [25]. Notably, in immature thymocytes, CHMP5 expression was downregulated by high‐affinity TCR ligands that cause negative selection but upregulated by low‐affinity TCR signaling and functioned to stabilize Bcl‐2 proteins and prevent postselection thymocyte apoptosis [25]. While our previous study established a critical role for CHMP5 in positive selection, investigation of CHMP5's function and its mechanism of regulation in post‐thymic T cells was precluded by the absence of mature αβ T cells in the periphery of experimental mice. Thus, CHMP5's specific function in mature T cells in the periphery is unknown. Using conditional and inducible cre recombinase genetic mouse models that selectively inactivate the *Chmp5* gene in mature post‐thymic T cells, we here uncover a critical requirement for CHMP5 in peripheral naïve T cell survival and homeostasis.

## 2 Results and Discussion

### 2.1 CHMP5 Is Required for Peripheral Naïve CD8^+^ T Cell Maintenance

To investigate the function of CHMP5 in post‐thymic T cells in vivo, we first inactivated the *Chmp5* gene using the *Cd8a*‐cre transgenic mice [26], where cre recombinase expression is targeted to mature (CD24^lo^TCRβ^hi^) CD8α single positive (SP) thymocytes and peripheral CD8α^+^ T cells by the *Cd8a* promoter plus E8_I_ enhancer [26, 27, 28]. Given very weak signals from the internal ribosomal entry site (IRES)‐GFP downstream of cre in this strain [26], we assessed cre activity by introducing *Cd8a*‐cre into a *Rosa26*‐encoded “*loxP*‐STOP‐*loxP*‐GFP” (*R26* ^LSL–GFP^) reporter mouse. Low GFP signals were detected in a few CD8 SP thymocytes, suggesting cre initiation in the SP thymocyte lineage, and in some CD11c^+^ cells, which can include a CD8α^+^ fraction [29]. Importantly, the majority (>80%) of GFP^+^ cells in spleens and lymph nodes (LN) of these mice were CD8^+^ T cells (Figure S1A,B), in line with *Cd8a*‐cre specific activity in post‐thymic peripheral CD8^+^ T cells [26].

We generated *Cd8a*‐cre^+^ *Chmp5* ^fl/fl^ (hereafter, *Chmp5* ^Cd8a^) mice, and these animals developed normally with no gross defects. Thymocyte cellularity and numbers of mature (CD24^lo^TCRβ^hi^) CD4 and CD8 SP thymocytes in *Chmp5* ^Cd8a^ mice were comparable to control (i.e., *Cd8a*‐cre^−^ *Chmp5* ^fl/fl^ or *Cd8a*‐cre^+^ *Chmp5* ^+/+^; *Chmp5* ^wt^) mice (Figure 1A,B). However, spleens and LNs of *Chmp5* ^Cd8a^ mice were markedly depleted of CD8^+^ T cells (Figure 1C), and this depletion was also evident in tissues including kidney, liver, and lungs of *Chmp5* ^Cd8a^ animals (Figure 1D). The few peripheral CD8^+^ T cells in *Chmp5* ^Cd8a^ mice were CD44^hi^ and expressed lower levels of TCRβ (Figure S1C,D), a “memory‐like” phenotype of T cells undergoing homeostatic proliferation driven by self‐antigen in lymphopenic hosts [13]. Interestingly, total CD4^+^ T cells were higher in spleens and LNs of *Chmp5* ^Cd8a^ mice (Figure 1C). This increase in CD4^+^ T cells could result from an increase in available IL‐7 [30], “surplused” due to CD8^+^ T cell depletion. Together, these results suggest a critical requirement for CHMP5 in the maintenance of peripheral naïve CD8^+^ T cells.

**FIGURE 1:**
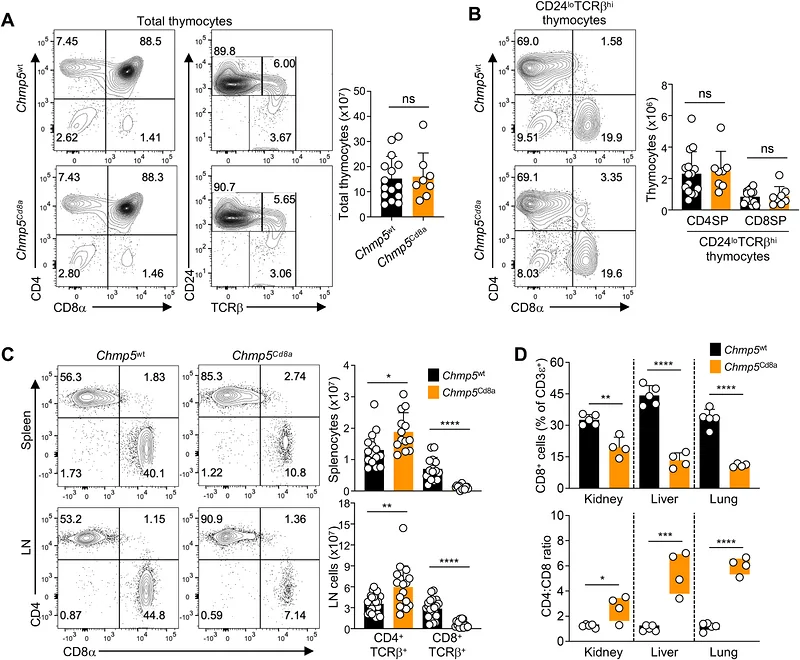
Essential role for CHMP5 in peripheral naïve CD8^+^ T cell maintenance. (A) Representative flow cytometry plots of CD4 versus CD8 expression (left), CD24 versus TCRβ expression (right), and absolute thymocyte numbers from the indicated mice. (B) Representative flow cytometry plots of CD4 versus CD8 expression and CD4 SP and CD8 SP thymocyte numbers within the CD24^lo^TCRβ^hi^ thymocyte population. (C) Representative flow cytometry plots of CD4 versus CD8 expression on splenic (top) and lymph node (bottom) and numbers of TCRβ^+^ subsets from the indicated mice. (D) CD8^+^ T cell frequency (top) and CD4:CD8 expression ratio (bottom) in CD3e^+^ T cells from kidney, liver, and lungs. Each data point is representative of a single mouse; unpaired Student's *t*‐test: ns, not significant. *p < 0.05; **p < 0.01; ***p < 0.001; ****p < 0.0001.

### 2.2 Cell‐Intrinsic Requirement for CHMP5 in Mature CD4^+^ and CD8^+^ T Cell Homeostasis

We next asked whether CHMP5 was also required for the maintenance of post‐thymic naïve CD4^+^ T cells by breeding *Chmp5* ^fl/fl^ mice to a hCD2‐cre transgenic mouse strain that expresses cre in late stages of thymocyte development [31]. In line with the expression of this hCD2‐cre strain in mature αβ T cells [31], cre‐induced YFP expression from an *R26* ^LSL–YFP^ reporter allele initiated in SP thymocytes and peaked (∼90%) in peripheral CD4^+^ and CD8^+^ T cells (Figure S1E,F). Thymi from these mice showed no differences in total or mature (CD24^lo^TCRβ^hi^) CD4 and CD8 SP thymocytes between hCD2‐Cre^+^ *Chmp5* ^fl/fl^ (hereafter, *Chmp5* ^hCD2^) and control (*Chmp5* ^wt^) mice (Figure 2A,B). However, analysis of *Chmp5* ^hCD2^ spleens and LNs revealed a significant decrease in αβ T cells, a reduction that was largely due to loss of CD8^+^ T cells, as CD4^+^ T cell numbers were comparable to those in *Chmp5* ^wt^ mice (Figure 2C,D). These CD4^+^ T cells, alongside the few CD8^+^ T cells in *Chmp5* ^hCD2^ mice, were CD44^hi^ with reduced expression of CD62L (Figure S1G–I), suggesting that they were T cells that expanded and adopted a “memory‐like” phenotype [13] as a result of T cell lymphopenia in these mice. Indeed, expression of the cell proliferation marker ki‐67 was significantly higher in peripheral CD4^+^ and CD8^+^ T cells from *Chmp5* ^hCD2^ mice (Figure 2E).

**FIGURE 2:**
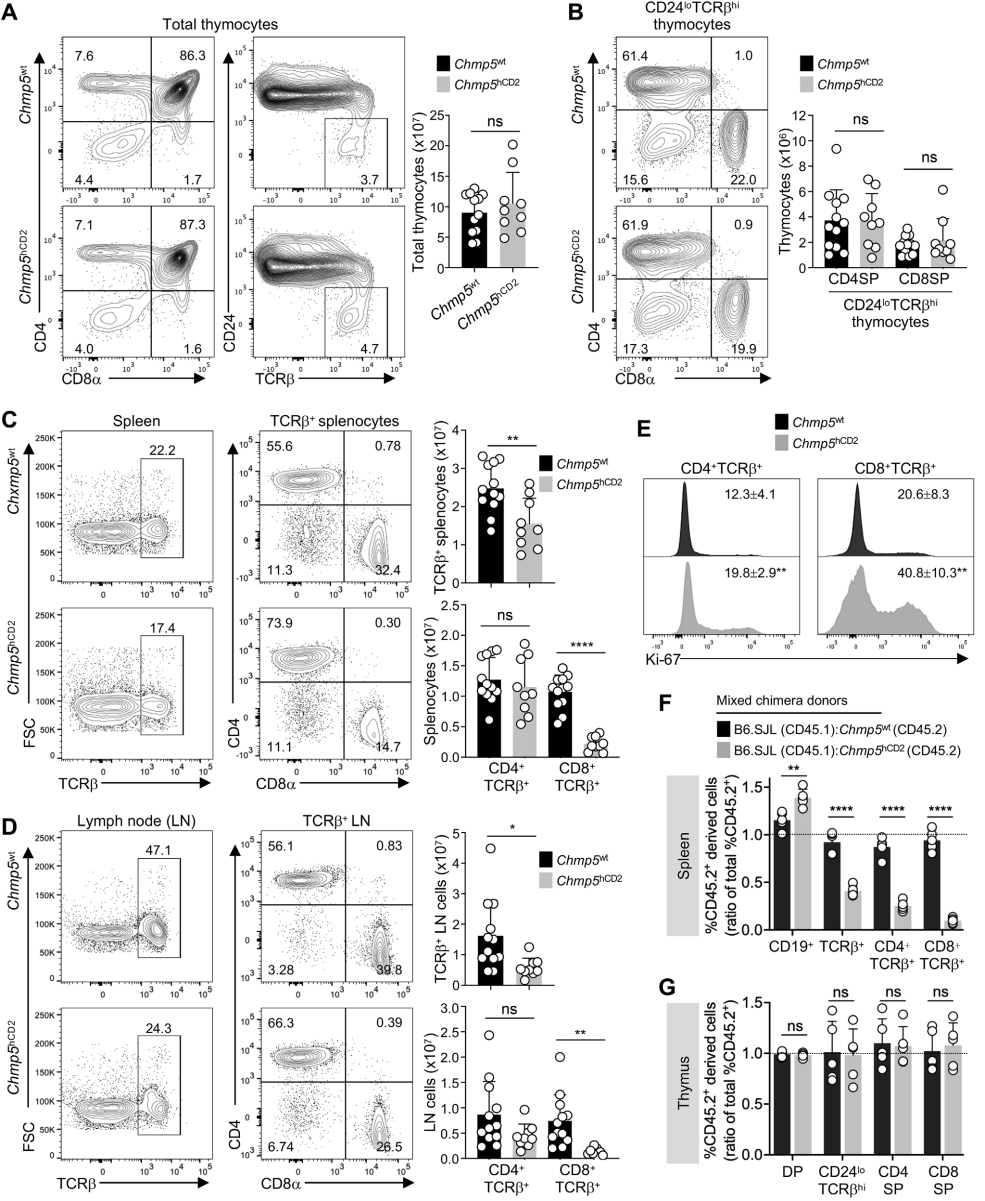
Cell‐intrinsic requirement for CHMP5 in peripheral CD4^+^ and CD8^+^ T cell homeostasis. (A) Representative flow cytometry plots of CD4 versus CD8 expression (left) and CD24 versus TCRβ expression (right) and thymocyte numbers from the indicated mice. (B) Representative flow cytometry plots of CD4 and CD8 expression on CD24^lo^TCRβ^hi^ thymocytes and absolute cell numbers of the indicated populations. (C, D) Representative flow cytometry plots of (TCRβ^+^) T cells (left) and cell numbers (right) of TCRβ^+^CD4^+^ and TCRβ^+^CD8^+^ cells from spleen (C) and lymph node (D). (E) Intracellular staining for Ki‐67 expression on splenic T cell subsets. (F, G) Proportion of CD45.2^+^ cells within the indicated immune cell subsets normalized to the total frequency of donor‐derived CD45.2^+^ cells in spleen (F) and thymus (G). Each data point represents a single mouse; unpaired students’ *t*‐test: ns, not significant; *p < 0.05; **p < 0.01; ***p < 0.001; ****p < 0.0001.

Despite the normal numbers of peripheral CD4^+^ T cells in these mice, loss of *Chmp5* resulted in specific and significant loss of phenotypically naïve (CD44^lo^CD62L^hi^) subsets in both CD4^+^ and CD8^+^ T cells in the periphery of *Chmp5* ^hCD2^ mice (Figure S1G–I). This implied a critical requirement for CHMP5 in the maintenance of peripheral naïve αβ T cells. To determine if this requirement was cell‐intrinsic or the result of potential cell‐extrinsic roles of CHMP5, we generated mixed bone marrow (BM) chimeras with CD45.2^+^ *Chmp5* ^wt^ or *Chmp5* ^hCD2^ mixed (1:1) with donor BM cells from wildtype B6.SJL (CD45.1^+^) mice. Donor BM cells were transplanted into lethally irradiated B6.SJL recipient mice (CD45.1^+^) and chimera mice were analyzed 8–9 weeks after transplantation. To account for differences in donor engraftment, we normalized donor‐derived CD45.2^+^ cell frequency within each population to the total frequency of CD45.2^+^ donor‐derived cells in the organ, where a ratio of ∼1.0 implies that the frequency of CD45.2^+^ within that population mirrors the frequency of donor‐derived CD45.2^+^ cells within that organ. As shown in Figure 2F, the contribution of *Chmp5* ^hCD2^ (CD45.2^+^) BM cells to both CD4^+^ and CD8^+^ TCRβ^+^ splenic cells was markedly diminished. By contrast, *Chmp5* ^hCD2^ BM donor cells contributed proportionally to B (CD19^+^) cell generation (Figure 2F). Importantly, the contribution of *Chmp5* ^hCD2^ BM cells to thymocyte subsets approximated their frequency the in total thymus (Figure 2G), which is consistent with the post‐thymic activity of the hCD2‐Cre. Taken together, these results document that CHMP5 supports post‐thymic naïve T cell homeostasis by a cell‐intrinsic mechanism.

### 2.3 CHMP5 Expression Is Posttranscriptionally Induced by TCR and IL‐7

To understand mechanisms regulating CHMP5 expression in T cells, we hypothesized that as key signals for naïve T cell homeostasis, TCR and IL‐7 regulate CHMP5 expression. Antibody crosslinking of CD3ε (TCR activation) upregulated CHMP5 proteins in naïve CD4^+^ and CD8^+^ T cells (Figure 3A) as well as in the parental Jurkat T cell line, but not in the Lck‐deficient line J.CaM1.6 [32] (Figure 3B). Co‐crosslinking of CD4 and CD8 coreceptors or CD28 further augmented CHMP5 expression (Figure 3A). CHMP5 was also induced by IL‐7 and IL‐2 (Figure 3A), in line with common γ‐chain cytokines promoting naïve T cell survival [33, 34, 35]. Notably, upregulation of CHMP5 by TCR and cytokines was independent of changes in *Chmp5* mRNA (Figure 3C), implying that these homeostatic signals posttranscriptionally regulate CHMP5 expression. Accordingly, TCR activation (by CD3ε antibody crosslinking) also upregulated expression of a heterologously expressed FLAG‐tagged CHMP5 that had been lentivirally transduced into Jurkat cells, and blocking new protein synthesis using cycloheximide suppressed this induction (Figure 3D). Thus, TCR and IL‐7 signals promote CHMP5 expression by a posttranscriptional mechanism, similar to its regulation in thymocytes [25]. Such posttranscriptional regulation might serve to ensure rapid modulation of CHMP5 abundance to effect naïve T cell homeostatic programs.

**FIGURE 3:**
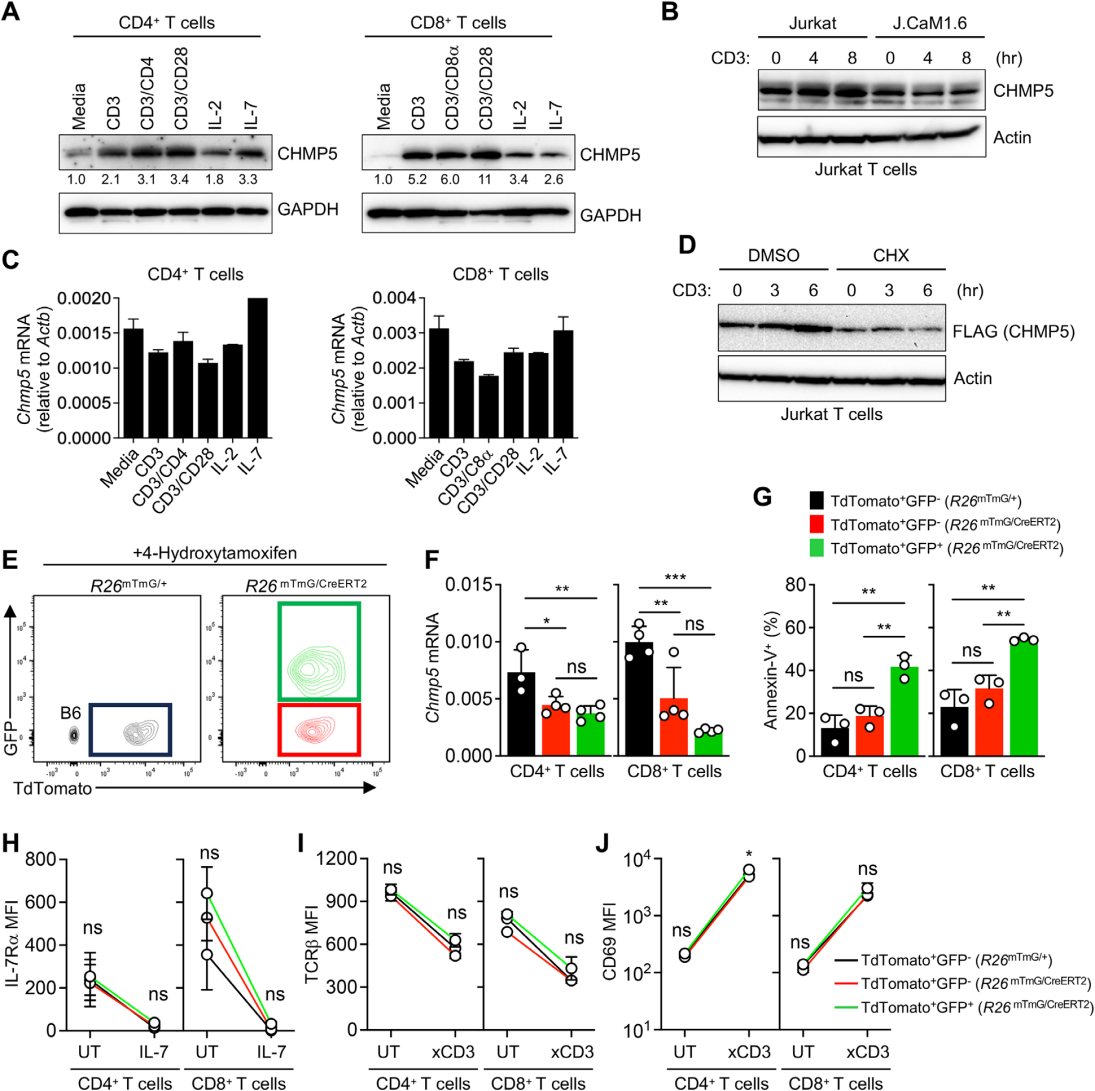
CHMP5 is upregulated by TCR and IL‐7 and prevents naïve T cell apoptosis. (A) Western blot of CHMP5 protein in naïve CD4^+^ or CD8^+^ T cells stimulated by anti‐CD3ε crosslinking or cytokines IL‐2 and IL‐7 for 18 h. Data are representative of 3 independent experiments. (B) Western blot of CHMP5 protein induction kinetics in Jurkat or J.CaM1.6 cells activated by anti‐CD3 antibody over 8 h. Data are representative of 3 independent experiments. (C) Real‐time qPCR of *Chmp5* mRNA expression (relative to *Actb*) in the naïve CD4^+^ and CD8^+^ T cells shown in panel (A). (D) Western blot of FLAG‐CHMP5 protein induction in Jurkat cells treated with vehicle (DMSO) or the protein synthesis inhibitor cycloheximide (CHX). Data are representative of three independent experiments. (E, F) Representative flow cytometry plots of tdTomato and GFP expression (E) and *Chmp5* transcripts (relative to *Actb*) in CD4^+^ and CD8^+^ T cells flow cytometry‐sorted (as shown in gates) from *R26* ^mTmG/CreERT2^ *Chmp5* ^fl/fl^ or control *R26* ^mTmG/+^ *Chmp5* ^fl/fl^ mice following 4‐hydroxytamoxifen treatment for 72 h. (G) Frequency of Annexin‐V^+^ CD4^+^ and CD8^+^ T cells following tamoxifen treatment. Each data point in (F) and (G) represents cells from a single mouse. One‐way ANOVA with Tukey's multiple comparison test: ns, not significant; *p < 0.05; **p < 0.01; *p* < 0.01; ***p < 0.001. (H) Geometric mean fluorescence intensity (MFI) of surface IL‐7Rα expression on the indicated tdTomato and GFP fractions of T cells untreated (UT) or treated with IL‐7 for 18 h at 37°C. (I, J) MFI of surface TCRβ (I) and CD69 (J) expression on T cell subsets untreated (UT) or treated overnight with anti‐CD3 crosslinking antibody (xCD3). Data are representative of three independent experiments. Two‐way ANOVA with Tukey's multiple comparisons test: ns, not significant; *p < 0.05.

### 2.4 CHMP5 Prevents Apoptosis in Post‐Thymic Mature αβ T Cells

To circumvent the depletion of post‐thymic naïve T cells upon CHMP5 deletion in our experimental mouse models and furnish *Chmp5*‐deleted T cells for mechanistic studies, we developed an inducible *Chmp5* deletion model. We specifically generated *Rosa26* ^mT^ ^mG/creERT2^ *Chmp5* ^fl/fl^ mice in which one *Rosa26* (*R26*) allele encoded the tamoxifen‐regulated cre‐ERT2 [36] and the other *R26* allele encoded a dual fluorescent reporter consisting of membrane tdTomato and cre‐inducible membrane GFP (“mTmG”) [37]. Purified naïve T cells from these mice were treated with tamoxifen for 72 h in IL‐7‐supplemented culture media (without TCR activation), which allowed for maintenance of their naïve state and survival. Compared with *Chmp5*‐sufficient control T cells (i.e., tdTomato^+^GFP^−^; from cre^−^ littermates or in the same culture), tamoxifen‐induced cre activity (i.e., tdTomato^+^GFP^+^ T cells) downregulated *Chmp5* mRNA (Figure 3E,F). Notably, loss of *Chmp5* was associated with elevated apoptosis (Annexin‐V^+^ staining) in both CD4^+^ and CD8^+^ T cells (Figure 3G), signifying that CHMP5 prevents apoptosis in mature αβ T cells.

The apoptosis resulting from CHMP5 depletion was not due to defects in IL‐7R signaling, as IL‐7Rα downregulation (Figure 3H), a consequence of IL‐7 signaling [33], was unchanged across both CD4^+^ and CD8^+^ CHMP5‐sufficient and deficient cells. Despite CHMP5's role in the ESCRT machinery [20, 21, 22] that mediates steady‐state and ligand‐induced membrane receptor turnover and downregulation [23, 38], *Chmp5* inactivation also did not affect steady‐state or activation‐induced downregulation of TCR or upregulation of CD69 (Figures 3I,J). To understand how loss of CHMP5 caused apoptosis, we next evaluated expression of Bcl‐2, a known pro‐survival factor stabilized by CHMP5 in thymocytes [25]. Surprisingly, Bcl‐2 protein expression was not disrupted in T cells after acute in vitro deletion of *Chmp5* (Figure S2A). Analysis of key pro‐survival (Bcl‐2 and Mcl‐1) and pro‐apoptotic (Bax) proteins in T cells from our *Chmp5* ^hCD2^ experimental mice also revealed modest to no changes in expression of these proteins in CHMP5‐deficient T cells (Figure S2B). This is in contrast to TCR‐signaled thymocytes lacking CHMP5, which displayed diminished expression of Bcl‐2 proteins [25], suggesting that CHMP5 prevents apoptosis independent of stabilizing Bcl‐2 family proteins in peripheral mature T cells.

Recent studies in periskeletal progenitor cells revealed that CHMP5 mitigated oxidative stress [39], which can be deleterious and cause apoptosis in T cells [40]. Thus, we considered whether CHMP5‐deficient T cells might be experiencing oxidative stress and used the CellROX reagent to quantify reactive oxygen species (ROS) in IL‐7‐cultured splenic T cells from our cre‐ERT2‐inducible *Chmp5* deletion experimental mice. After 48 h of tamoxifen treatment, we found higher levels of ROS in T cells with recombined *Chmp5* gene (i.e., GFP^+^ cells) compared with T cells with wild‐type *Chmp5* allele, albeit this was only significant in CD8^+^ T cells (Figure S2C). We next assayed ROS levels in ex vivo T cells from our other CHMP5‐deficient experimental mouse models. Compared with CHMP5‐sufficient counterparts from *Chmp5* ^wt^ mice, we found higher ROS levels in *ex vivo* splenic CD8^+^ T cells from *Chmp5* ^hCD2^ mice (Figure S2D) and from CD8^+^ T cells derived from *Chmp5* ^hCD2^ BM chimera donors (Figure S2E). ROS levels were comparable between CHMP5‐sufficient and CHMP5‐deficient thymocytes (Figure S2D,E), consistent with the post‐thymic activity of the hCD2‐Cre. Interestingly, we observed no difference in cellular ROS between ex vivo splenic CD4^+^ T cells from *Chmp5* ^wt^ and *Chmp5* ^hCD2^ genetic backgrounds (Figure S2D,E). Taken together, these results suggest that CHMP5 might function to promote peripheral T cell homeostasis by preventing oxidative stress in T cells, which can potentially account for increased apoptosis in CHMP5‐deficient T cells.

Collectively, our results indicate that, beyond promoting the survival of TCR‐signaled thymocytes [25], sustained expression of CHMP5 is required for post‐thymic αβ T cell homeostasis in the periphery. This requirement is at least in part because CHMP5 functions to prevent apoptosis in peripheral T cells. Compared with CD8^+^ T cells, there appeared to be a less stringent requirement for CHMP5 in CD4^+^ T cells, whose numbers in *Chmp5* ^hCD2^ mice were largely comparable to CHMP5‐sufficient littermate controls. On one hand, this discrepancy might suggest a redundant role for CHMP5 in CD4^+^ T cells or reflect clonal composition differences between these T cell subsets. Clonal size, where less abundant clones survive longer than more abundant clones, governs T cell expansion during lymphopenia [41, 42]. Alternatively, as the CD44^hi^ phenotype and higher ki‐67^+^ cell frequency (Figure 2C,D; Figure S1G–I) suggest that residual T cells in *Chmp5* ^hCD2^ mice are lymphopenia‐induced “memory‐like” cells [13], the discrepancy could also indicate that CD44^hi^CD4^+^ T cells are not dependent on IL‐7 as CD44^hi^CD8^+^ T cells are [43]. Nevertheless, both CD8^+^ and CD4^+^ T cells lacking CHMP5 were outcompeted in the presence of wildtype T cells in mixed BM chimeras, indicating that loss of CHMP5 impaired the fitness of both αβ T cell subsets in the presence of CHMP5‐sufficient cells. We note that our findings of high CD44^hi^ T cell numbers in *Chmp5* ^hCD2^ mice contrasted with findings from *Cd4*‐cre‐deleted *Chmp5* mice, which were severely depleted of all mature SP thymocytes as well as peripheral CD4^+^ and CD8^+^ T cells [25]. This is likely attributable to the consequence of CHMP5 loss in DP thymocytes in the *Cd4*‐cre model, impairing development of TCR‐signaled thymocytes [25], the precursors of mature αβ T cells [2].

### 2.5 Data Limitations and Perspectives

The prosurvival function of CHMP5 described here reinforces growing evidence of an antiapoptotic function of CHMP5 in various mammalian cell types [25, 44, 45, 46, 47]. CHMP5 deficiency in peripheral αβ T cells did not appear to alter canonical ESCRT machinery‐driven cellular processes, including surface receptor flux (Figure 3H–J). Thus, in line with evidence that individual ESCRT proteins have evolved roles unrelated to membrane remodeling in metazoans [48, 49], it is tempting to speculate that a “noncanonical” activity might underlie CHMP5's function in mature peripheral αβ T cells. Indeed, we and others have shown that CHMP5 can regulate cellular homeostasis and function by promoting client protein stability [25, 50] or modulating the activity of transcriptional coactivators like BRD4 and p300 to drive gene expression [51]. Nevertheless, further studies are needed to determine how and whether CHMP5 mechanistically regulates apoptosis and overall naïve T cell homeostasis by similar or different posttranslational and transcriptional activities. Furthermore, the basis of the distinct impact of CHMP5 deficiency on the CD4^+^ and CD8^+^ T cell phenotype and numbers in vivo, as well as the differential effect on the redox state of these T cell subsets, remains to be determined.

### 2.6 Concluding Remarks

This study uncovers CHMP5 as a TCR‐ and IL‐7‐induced protein critical for peripheral naïve T cell survival and highlights a previously unrecognized mechanism in which CHMP5 is required to dampen oxidative stress and prevent apoptosis in peripheral naïve T cells. That TCR and IL‐7 signals upregulated CHMP5 protein, and not its mRNA, indicates a novel posttranscriptional regulatory mechanism of naïve T cell survival that would not have been captured by transcriptomic analysis alone. Finally, because naïve T cell survival requires that IL‐7 signals be intermittently interrupted by TCR signals [52, 53], we propose that CHMP5 upregulation, independently by either TCR or IL‐7, prevents complete disruption and ensures continuous expression of the homeostatic program required for naïve T cell maintenance.

## 3 Materials and Methods

### 3.1 Mice

C57BL/6J, B6;129‐*Gt(ROSA)26Sor^2Sho^*/J (R26R‐EGFP) [54], B6.129‐*Gt(ROSA)26Sor^1(c^* ^re/ERT2)Tyj^/J (RosaERT2‐cre) [36], B6.129X1‐*Gt(ROSA)26Sor* ^1(EYFP)Cos^/J (R26R‐EYFP), *Gt(ROSA)26Sor^4(ACTB–tdTomato,–EGFP)Luo^*/J (mTmG) [37], C57BL/6‐Tg(Cd8a‐cre)1Itan/J (Cd8a‐cre) [26], and bone marrow chimera recipient B6.SJL‐*Ptprc^a^ Pepc^b^*/BoyJ (B6.SJL) mice were purchased from Jackson Laboratory (Bar Harbor, Maine, USA). C57BL/6‐Tg(CD2‐cre)1Lov/J (human (h) CD2‐cre) mice [31] were provided by Dr. Paul Love (National Institutes of Health, USA). *Chmp5* ^fl/fl^ mice (with loxP‐flanked exons 3 through 7 of the *Chmp5* gene) were previously described [55]. Animal procedures were approved by the Case Western Reserve University (Ohio, USA) Institutional Animal Care and Use Committee (IACUC) or the NCI Animal Care and Use Committee (ACUC) in accordance with federal laws and standards. All components of the intramural NIH ACU program are accredited by AAALAC International. Except otherwise indicated, mice were housed in specific‐pathogen‐free facilities and analyzed between 8 and 16 weeks of age.

### 3.2 Bone Marrow Chimeras

Bone marrow (BM) was obtained by flushing tibia and femur marrow cavities with ACK red cell lysis buffer (Fisher Scientific #A1049201), before washing with PBS and depletion of T‐cells using a CD3ɛ microbead kit (Miltenyi Biotec #130‐094‐973). To assess the contributions of non‐cell‐intrinsic mechanisms to peripheral T cell depletion in CHMP5‐deficiency, 1:1 mixed bone marrow chimeras of wildtype CD45.1^+^ cells (1 × 10^6^ cells) and either hCD2Cre^+^Chmp5^fl/fl^ YFP^+^ (Chmp5hCD2) or hCD2Cre^+^Chmp5^+/+^ YFP^+^ (Chmp5wt) cells (1 × 10^6^ cells) were transferred into lethally irradiated (1000 rads) wildtype CD45.1^+^ irradiated host animals. Chimeras were analyzed at 8–12 weeks after transplantation.

### 3.3 Primary T Cell Isolation, Cell Lines, and Culture

Thymus, lymph node, and spleen were mechanically dissociated to obtain single‐cell suspensions. Splenocytes were treated with ACK buffer (ThermoFisher # A1049201) to lyse red blood cells. To obtain tissue T cells, lung, liver, and kidney were perfused and then subjected to mechanical and enzymatic dissociation as described [56, 57, 58]. Single‐cell suspensions were passed through 40‐70‐µm strainers and resuspended in complete RPMI (RPMI 1640 supplemented with 2 mM L‐glutamine, 1 mM sodium pyruvate, 10 mM HEPES [pH 7.4], 1× MEM nonessential amino acids, 50 IU/mL penicillin, 50 µg/mL streptomycin, 55 µM β‐mercaptoethanol, and 10% FBS). Jurkat (#TIB‐152) and the mutant Jurkat line J.CaM1.6 (#CRL‐2063) [32] were purchased from ATCC (Manassas, VA) and maintained in complete RPMI. All cells were cultured at 37°C in 5% CO2 incubators.

### 3.4 In Vitro Stimulation and Drug Treatments

For primary T cell stimulation, plates were coated overnight at 4°C with 5 µg/mL anti‐CD3 (BD Biosciences #553057) alone or in combination with 1 µg/mL anti‐CD28 (BD #553294), anti‐CD4 (BD Biosciences #553726), or anti‐CD8α (BD Biosciences #555363) in PBS. Recombinant murine IL‐7 (PeproTech #217‐17‐250UG) was added to culture conditions at a concentration of 10 ng/mL and recombinant IL‐2 (PeproTech #212‐12‐250UG) at a concentration of 50 U/mL. For ERT2cre‐mediated deletion in vitro, cells were treated with 2 µM 4‐hydroxytamoxifen (Millipore‐Sigma #H6278) for 72 h in the presence of IL‐7. Where indicated, cycloheximide (CHX; Millipore‐Sigma #01810) was used at a concentration of 50 µg/mL for protein synthesis inhibition.

### 3.5 Flow Cytometry Analysis and Cell Sorting

Single‐cell suspensions were washed with flow cytometry buffer (PBS, 2% FBS, and 1 mM EDTA) and incubated with fluorochrome‐conjugated antibodies (Table S1) for 45 min at 4°C in the dark. After washing, stained cells were resuspended in flow cytometry buffer containing DAPI (0.5 µg/mL; Life Technologies) to exclude dead cells. Apoptosis was detected using Annexin V‐APC (BioLegend #640941) and Annexin V Binding Buffer (BioLegend #422201). CellROX staining was performed using CellROX Deep Red (Invitrogen #C10422) according to the manufacturer's instructions. For intracellular staining, a fixable live‐dead stain (LIVE/DEADTM Fixable Blue Dead Cell Stain Kit, Thermo‐Fisher #L34962) was included in the surface staining cocktail; staining was performed as above. Intracellular staining of thymocytes and peripheral T‐cells was performed using the eBioscience Foxp3/transcription factor staining buffer set (Thermo #00‐5523‐00) according to the manufacturer's instructions. Briefly, cells were fixed for 30 min at RT, washed twice, stained for intracellular proteins in 1x permeabilization buffer for 3 min at room temperature, and then washed further before analysis. Cell sorting was carried out on a BD FACS Aria‐SORP instrument, and flow cytometry analysis was performed on the BD LSR Fortessa or FACSymphony A5. Data analysis was done using FlowJo 10 (Becton Dickinson).

### 3.6 RNA Isolation and Quantitative Real‐Time PCR

Total RNA was extracted from all cells using TRIzol reagent (ThermoFisher, #15596026), and 100–500 ng of RNA was reverse transcribed using the high‐capacity cDNA reverse transcription kit (ThermoFisher, #4368814). Real‐time qPCR was performed using Fast SYBR Green Master Mix (Applied Biosystems, #43‐856‐12) on a QuantStudio 6 Flex real‐time PCR system (Applied Biosystems, #4485691). Gene expression levels were calculated using the ΔCT method, where ΔCT = CT(gene of interest) − CT(housekeeping gene), and results were expressed as 2^(−ΔCT). These primers were used: β‐actin (*Actb*): forward/reverse: 5′‐CATTGCTGACAGGATGCAGAAGG‐3′, 5′‐TGCTGGAAGGTGGACAGTGAGG‐3′; *Chmp5*: 5′‐GATGAGAGAGGGTCCTGCTAAG‐3′, reverse: 5′‐CAGGTTGTCTCGCTGTTGCTCA‐3′.

### 3.7 Protein Isolation, Electrophoresis and Immunoblotting

Cells were washed with ice‐cold PBS by centrifugation at 5000 rpm for 5 min at 4°C. Cell pellets were either stored at −80°C or lysed immediately. Cells were lysed in RIPA buffer (25 mM Tris‐HCl pH 7.6, 150 mM NaCl, 1% NP‐40, 1% sodium deoxycholate, and 0.1% SDS) containing 1× PhosSTOP (Sigma‐Aldrich #4906837001) and 1× EDTA‐free Protease Inhibitor Cocktail (Sigma‐Aldrich #4693159001). Lysates were clarified by centrifugation at 13,000 rpm for 12 min at 4°C. Protein concentrations were quantified using the Pierce BCA protein assay kit (ThermoFisher #23225). For western blotting, 10–40 µg of total protein was diluted in NuPAGE LDS sample buffer (ThermoFisher #NP0007) containing sample reducing agent (ThermoFisher #NP0009). After boiling at 70°C for 10 min, samples were run on NuPAGE 4%–12% Bis‐Tris Protein Gels (ThermoFisher #NP0335BOX), transferred to a PVDF membrane (EMD Millipore #IPVH00005), and probed as described [59] using primary and secondary antibodies listed in Table S2.

### 3.8 Data Analysis and Statistics

All experiments were independently repeated at least three times, and no data points were excluded from analysis. Unless otherwise indicated, comparisons between groups were performed using two‐tailed, unpaired Student's *t*‐tests, and differences were considered statistically significant at *p* < 0.05. Significance levels are denoted as follows: *p < 0.05; **p < 0.01; ***p < 0.001; and ****p < 0.0001. All statistical analyses were performed using GraphPad Prism version 10.0.

## Author Contributions

Conceptualization: S.A., J.T., and K.U‐W. Investigation: J.T., K.U‐W., E.B., Y‐C. L, J‐H.S., and S.A. Data curation/formal analysis: J.T., K.U‐W., and S.A. Project administration/funding: S.A. Writing – original draft, review, editing, and revision: J.T. and S.A. with input from all authors.

## Disclaimer

The contributions of the NIH authors were made as part of their official duties as NIH federal employees, are in compliance with agency policy requirements, and are considered Works of the United States Government. However, the findings and conclusions presented in this paper are those of the authors and do not necessarily reflect the views of the NIH or the U.S. Department of Health and Human Services.

## Ethics Statement

All animal experiments were initiated only after approval by the Case Western Reserve University (Cleveland, Ohio, USA) Institutional Animal Care and Use Committee (IACUC) or the National Cancer Institute (Bethesda, Maryland, USA) Animal Care and Use Committee (ACUC) in accordance with federal laws and standards. All components of the intramural National Institutes of Health (NIH) ACUC program are accredited by AAALAC International. Animals were handled and housed under humane conditions and experiments performed as approved in the above protocols.

## Conflicts of Interest

All authors declare no conflicts of interest.

## Supporting information




**Supporting File 1**: eji70310‐sup‐0001‐FigureS1‐S2.pdf.

**Supporting File 2**: eji70310‐sup‐0002‐TableS1‐S2.xlsx.

## Data Availability

The data that support the findings of this study are available from the corresponding author upon reasonable request.

## References

[1] L. Klein , B. Kyewski , P. M. Allen , and K. A. Hogquist , “Positive and Negative Selection of the T Cell Repertoire: What Thymocytes See (and Don't See),” Nature Reviews Immunology 14 (2014): 377–391, 10.1038/nri3667. PMC4757912 24830344

[2] A. Singer , S. Adoro , and J. H. Park , “Lineage Fate and Intense Debate: Myths, Models and Mechanisms of CD4‐ Versus CD8‐Lineage Choice,” Nature Reviews Immunology 8 (2008): 788–801, 10.1038/nri2416. PMC2760737 18802443

[3] T. Hapuarachchi , J. Lewis , and R. E. Callard , “A Mechanistic Model for Naive CD4 T Cell Homeostasis in Healthy Adults and Children,” Frontiers in Immunology 4 (2013): 366, 10.3389/fimmu.2013.00366. PMC3822783 24273540

[4] S. Rane , T. Hogan , E. Lee , B. Seddon , and A. J. Yates , “Towards a Unified Model of Naive T Cell Dynamics Across the Lifespan,” eLife 11 (2022): e78168, 10.7554/eLife.78168. PMC9348855 35678373

[5] T. Kawabe , J. Yi , and J. Sprent , “Homeostasis of Naive and Memory T Lymphocytes,” Cold Spring Harbor Perspectives in Biology 13 (2021): a037879, 10.1101/cshperspect.a037879. PMC8411951 33753403

[6] K. Takada and S. C. Jameson , “Naive T Cell Homeostasis: From Awareness of Space to a Sense of Place,” Nature Reviews Immunology 9 (2009): 823–832, 10.1038/nri2657. 19935802

[7] U. von Freeden‐Jeffry , P. Vieira , L. A. Lucian , T. McNeil , S. E. Burdach , and R. Murray , “Lymphopenia in Interleukin (IL)‐7 Gene‐Deleted Mice Identifies IL‐7 as a Nonredundant Cytokine,” Journal of Experimental Medicine 181 (1995): 1519–1526, 10.1084/jem.181.4.1519. PMC2191954 7699333

[8] K. S. Schluns , W. C. Kieper , S. C. Jameson , and L. Lefrancois , “Interleukin‐7 Mediates the Homeostasis of Naïve and Memory CD8 T Cells in Vivo,” Nature Immunology 1 (2000): 426–432, 10.1038/80868. 11062503

[9] J. J. Peschon , P. J. Morrissey , K. H. Grabstein , et al., “Early Lymphocyte Expansion is Severely Impaired in Interleukin 7 Receptor‐Deficient Mice,” Journal of Experimental Medicine 180 (1994): 1955–1960, 10.1084/jem.180.5.1955. PMC2191751 7964471

[10] B. Seddon and R. Zamoyska , “TCR Signals Mediated by Src Family Kinases are Essential for the Survival of Naive T Cells,” Journal of Immunology 169 (2002): 2997–3005, 10.4049/jimmunol.169.6.2997. 12218114

[11] B. Polic , D. Kunkel , A. Scheffold , and K. Rajewsky , “How αβ T Cells deal With Induced TCRα Ablation,” Proceedings of the National Academy of Sciences 98 (2001): 8744–8749, 10.1073/pnas.141218898. PMC37506 11447257

[12] B. Martin , C. Bourgeois , N. Dautigny , and B. Lucas , “On the Role of MHC Class II Molecules in the Survival and Lymphopenia‐Induced Proliferation of Peripheral CD4^+^ T Cells,” Proceedings of the National Academy of Sciences 100 (2003): 6021–6026, 10.1073/pnas.1037754100. PMC156319 12719530

[13] J. Sprent and C. D. Surh , “Normal T Cell Homeostasis: The Conversion of Naive Cells Into Memory‐Phenotype Cells,” Nature Immunology 12 (2011): 478–484, 10.1038/ni.2018. PMC3434123 21739670

[14] M. Pellegrini , P. Bouillet , M. Robati , G. T. Belz , G. M. Davey , and A. Strasser , “Loss of Bim Increases T Cell Production and Function in Interleukin 7 Receptor–Deficient Mice,” Journal of Experimental Medicine 200 (2004): 1189–1195, 10.1084/jem.20041328. PMC2211860 15504823

[15] A. R. Khaled , W. Q. Li , J. Huang , et al., “Bax Deficiency Partially Corrects Interleukin‐7 Receptor α Deficiency,” Immunity 17 (2002): 561–573, 10.1016/s1074-7613(02)00450-8. 12433363

[16] K. Akashi , M. Kondo , U. von Freeden‐Jeffry , R. Murray , and I. L. Weissman , “Bcl‐2 Rescues T Lymphopoiesis in Interleukin‐7 Receptor–Deficient Mice,” Cell 89 (1997): 1033–1041, 10.1016/s0092-8674(00)80291-3. 9215626

[17] E. Maraskovsky , L. A. O'Reilly , M. Teepe , L. M. Corcoran , J. J. Peschon , and A. Strasser , “Bcl‐2 Can Rescue T Lymphocyte Development in Interleukin‐7 Receptor–Deficient Mice but Not in Mutant rag‐1^−/−^ Mice,” Cell 89 (1997): 1011–1019, 10.1016/s0092-8674(00)80289-5. 9215624

[18] J. R. Köhler , “Mos10 (Vps60) is Required for Normal Filament Maturation in *Saccharomyces cerevisiae* ,” Molecular Microbiology 49 (2003): 1267–1285, 10.1046/j.1365-2958.2003.03556.x. 12940986

[19] A. Kranz , A. Kinner , and R. Kölling , “A Family of Small Coiled‐Coil–Forming Proteins Functioning at the Late Endosome in Yeast,” Molecular Biology of the Cell 12 (2001): 711–723, 10.1091/mbc.12.3.711. PMC30975 11251082

[20] I. F. Azmi , B. A. Davies , J. Xiao , M. Babst , Z. Xu , and D. J. Katzmann , “ESCRT‐III Family Members Stimulate Vps4 ATPase Activity Directly or via Vta1,” Developmental Cell 14 (2008): 50–61, 10.1016/j.devcel.2007.10.021. 18194652

[21] M. Bajorek , E. Morita , J. J. Skalicky , S. G. Morham , M. Babst , and W. I. Sundquist , “Biochemical Analyses of Human IST1 and Its Function in Cytokinesis,” Molecular Biology of the Cell 20 (2009): 1360–1373, 10.1091/mbc.e08-05-0475. PMC2649257 19129479

[22] H. T. H. Tsang , J. W. Connell , S. E. Brown , A. Thompson , E. Reid , and C. M. Sanderson , “A Systematic Analysis of human CHMP Protein Interactions: Additional MIT Domain‐Containing Proteins Bind to Multiple Components of the Human ESCRT III Complex,” Genomics 88 (2006): 333–346, 10.1016/j.ygeno.2006.04.003. 16730941

[23] D. J. Katzmann , G. Odorizzi , and S. D. Emr , “Receptor Downregulation and Multivesicular‐Body Sorting,” Nature Reviews Molecular Cell Biology 3 (2002): 893–905, 10.1038/nrm973. 12461556

[24] M. Vietri , M. Radulovic , and H. Stenmark , “The Many Functions of ESCRTs,” Nature Reviews Molecular Cell Biology 21 (2020): 25–42, 10.1038/s41580-019-0177-4. 31705132

[25] S. Adoro , K. H. Park , S. E. Bettigole , et al., “Post‐Translational Control of T Cell Development by the ESCRT Protein CHMP5,” Nature Immunology 18 (2017): 780–790, 10.1038/ni.3764. 28553951

[26] Y. Maekawa , Y. Minato , C. Ishifune , et al., “Notch2 Integrates Signaling by the Transcription Factors RBP‐J and CREB1 to Promote T Cell Cytotoxicity,” Nature Immunology 9 (2008): 1140–1147, 10.1038/ni.1649. 18724371

[27] S. Adoro , B. Erman , S. D. Sarafova , et al., “Targeting CD4 Coreceptor Expression to Postselection Thymocytes Reveals That CD4/CD8 Lineage Choice Is Neither Error‐Prone nor Stochastic,” Journal of Immunology 181 (2008): 6975–6983, 10.4049/jimmunol.181.10.6975. PMC7217309 18981117

[28] W. Ellmeier , M. J. Sunshine , K. Losos , F. Hatam , and D. R. Littman , “An Enhancer That Directs Lineage‐Specific Expression of CD8 in Positively Selected Thymocytes and Mature T Cells,” Immunity 7 (1997): 537–547, 10.1016/s1074-7613(00)80375-1. 9354474

[29] K. Shortman and W. R. Heath , “The CD8^+^ Dendritic Cell Subset,” Immunological Reviews 234 (2010): 18–31, 10.1111/j.0105-2896.2009.00870.x. 20193009

[30] C. N. Miller , D. J. Hartigan‐O'Connor , M. S. Lee , et al., “IL‐7 Production in Murine Lymphatic Endothelial Cells and Induction in the Setting of Peripheral Lymphopenia,” International Immunology 25 (2013): 471–483, 10.1093/intimm/dxt012. PMC3720065 23657000

[31] M. S. Vacchio , L. Wang , N. Bouladoux , et al., “A ThPOK‐LRF Transcriptional Node Maintains the Integrity and Effector Potential of Post‐Thymic CD4^+^ T Cells,” Nature Immunology 15 (2014): 947–956, 10.1038/ni.2960. PMC4251968 25129370

[32] D. B. Straus and A. Weiss , “Genetic Evidence for the Involvement of the Lck Tyrosine Kinase in Signal Transduction Through the T Cell Antigen Receptor,” Cell 70 (1992): 585–593, 10.1016/0092-8674(92)90428-f. 1505025

[33] J.‐H. Park , Q. Yu , B. Erman , et al., “Suppression of IL7Rα Transcription by IL‐7 and Other Prosurvival Cytokines,” Immunity 21 (2004): 289–302, 10.1016/j.immuni.2004.07.016. 15308108

[34] J. C. Rathmell , E. A. Farkash , W. Gao , and C. B. Thompson , “IL‐7 Enhances the Survival and Maintains the Size of Naive T Cells,” Journal of Immunology 167 (2001): 6869–6876, 10.4049/jimmunol.167.12.6869. 11739504

[35] Y. Rochman , R. Spolski , and W. J. Leonard , “New Insights Into the Regulation of T Cells by γc Family Cytokines,” Nature Reviews Immunology 9 (2009): 480–490, 10.1038/nri2580. PMC2814538 19543225

[36] A. Ventura , D. G. Kirsch , M. E. McLaughlin , et al., “Restoration of p53 Function Leads to Tumour Regression in Vivo,” Nature 445 (2007): 661–665, 10.1038/nature05541. 17251932

[37] M. D. Muzumdar , B. Tasic , K. Miyamichi , L. Li , and L. Luo , “A Global Double‐Fluorescent Cre Reporter Mouse,” Genesis 45 (2007): 593–605, 10.1002/dvg.20335. 17868096

[38] S. Vardhana , K. Choudhuri , R. Varma , and M. L. Dustin , “Essential Role of Ubiquitin and TSG101 Protein in Formation and Function of the Central Supramolecular Activation Cluster,” Immunity 32 (2010): 531–540, 10.1016/j.immuni.2010.04.005. PMC2905630 20399684

[39] F. Zhang , Y. Wang , L. Zhang , et al., “The ESCRT Protein CHMP5 Restricts Bone Formation by Controlling Endolysosome‐Mitochondrion‐Mediated Cell Senescence,” eLife 13 (2025): RP101984, 10.7554/eLife.101984. PMC12234009 40622747

[40] D. A. Hildeman , T. Mitchell , T. K. Teague , et al., “Reactive Oxygen Species Regulate Activation‐Induced T Cell Apoptosis,” Immunity 10 (1999): 735–744, 10.1016/s1074-7613(00)80072-2. 10403648

[41] J. Hataye , J. J. Moon , A. Khoruts , C. Reilly , and M. K. Jenkins , “Naïve and Memory CD4^+^ T Cell Survival Controlled by Clonal Abundance,” Science 312 (2006): 114–116, 10.1126/science.1124228. 16513943

[42] J. J. Obar , K. M. Khanna , and L. Lefrancois , “Endogenous Naive CD8^+^ T Cell Precursor Frequency Regulates Primary and Memory Responses to Infection,” Immunity 28 (2008): 859–869, 10.1016/j.immuni.2008.04.010. PMC2836785 18499487

[43] J. T. Tan , B. Ernst , W. C. Kieper , E. LeRoy , J. Sprent , and C. D. Surh , “Interleukin (IL)‐15 and IL‐7 Jointly Regulate Homeostatic Proliferation of Memory Phenotype CD8^+^ Cells but Are Not Required for Memory Phenotype CD4^+^ Cells,” Journal of Experimental Medicine 195 (2002): 1523–1532, 10.1084/jem.20020066. PMC2193564 12070280

[44] W. Gao , R. Liu , K. Huang , et al., “CHMP5 Attenuates Osteoarthritis via Inhibiting Chondrocyte Apoptosis and Extracellular Matrix Degradation: Involvement of NF‐κB Pathway,” Molecular Medicine 30 (2024): 55, 10.1186/s10020-024-00819-6. PMC11046779 38664616

[45] M. Shahmoradgoli , O. Mannherz , F. Engel , et al., “Antiapoptotic Function of Charged Multivesicular Body Protein 5: A Potentially Relevant Gene in Acute Myeloid Leukemia,” International Journal of Cancer 128 (2011): 2865–2871, 10.1002/ijc.25632. 20734392

[46] H. Wang , J. Liu , F. Wang , et al., “The Role of Charged Multivesicular Body Protein 5 in Programmed Cell Death in Leukemic Cells,” Acta Biochimica et Biophysica Sinica 45 (2013): 383–390, 10.1093/abbs/gmt028. 23619569

[47] Y. Yang , M. Wang , Y. Y. Zhang , S. Z. Zhao , and S. Gu , “The Endosomal Sorting Complex Required for Transport Repairs the Membrane to Delay Cell Death,” Frontiers in Oncology 12 (2022): 1007446, 10.3389/fonc.2022.1007446. PMC9622947 36330465

[48] T. Slagsvold , K. Pattni , L. Malerod , and H. Stenmark , “Endosomal and Non‐Endosomal Functions of ESCRT Proteins,” Trends in Cell Biology 16 (2006): 317–326, 10.1016/j.tcb.2006.04.004. 16716591

[49] A. Ray , Y. Rai , and M. S. Inamdar , “The Endosomal Sorting Complex, ESCRT, Has Diverse Roles in Blood Progenitor Maintenance, Lineage Choice and Immune Response,” Biology Open 13 (2024): bio060412, 10.1242/bio.060412. PMC11212638 38828842

[50] M. B. Greenblatt , K. H. Park , H. Oh , et al., “CHMP5 Controls Bone Turnover Rates by Dampening NF‐κB Activity in Osteoclasts,” Journal of Experimental Medicine 212 (2015): 1283–1301, 10.1084/jem.20150407. PMC4516796 26195726

[51] K. Umphred‐Wilson , S. Ratnayake , Q. Tang , et al., “The ESCRT Protein CHMP5 Promotes T Cell Leukemia by Enabling BRD4‐p300‐Dependent Transcription,” Nature Communications 16 (2025): 4133, 10.1038/s41467-025-59504-9. PMC12049546 40319015

[52] M. Y. Kimura , L. A. Pobezinsky , T. I. Guinter , et al., “IL‐7 Signaling Must be Intermittent, not Continuous, During CD8^+^ T Cell Homeostasis to Promote Cell Survival Instead of Cell Death,” Nature Immunology 14 (2013): 143–151, 10.1038/ni.2494. PMC3552087 23242416

[53] P. Koenen , S. Heinzel , E. M. Carrington , et al., “Mutually Exclusive Regulation of T Cell Survival by IL‐7R and Antigen Receptor‐Induced Signals,” Nature Communications 4 (2013): 1735, 10.1038/ncomms2719. PMC3644093 23591902

[54] X. Mao , Y. Fujiwara , A. Chapdelaine , H. Yang , and S. H. Orkin , “Activation of EGFP Expression by Cre‐Mediated Excision in a New ROSA26 Reporter Mouse Strain,” Blood 97 (2001): 324–326, 10.1182/blood.v97.1.324. 11133778

[55] J.‐H. Shim , C. Xiao , M. S. Hayden , et al., “CHMP5 is Essential for Late Endosome Function and Down‐Regulation of Receptor Signaling During Mouse Embryogenesis,” Journal of Cell Biology 172 (2006): 1045–1056, 10.1083/jcb.200509041. PMC2063762 16567502

[56] K. G. Blom , M. Rahman Qazi , J. B. Noronha Matos , B. D. Nelson , J. W. DePierre , and M. Abedi‐Valugerdi , “Isolation of Murine Intrahepatic Immune Cells Employing a Modified Procedure for Mechanical Disruption and Functional Characterization of the B, T and Natural Killer T Cells Obtained,” Clinical and Experimental Immunology 155 (2009): 320–329, 10.1111/j.1365-2249.2008.03815.x. PMC2675264 19040612

[57] N. P. Rudemiller and S. D. Crowley , “Characterization and Functional Phenotyping of Renal Immune Cells via Flow Cytometry,” Methods in Molecular Biology 1614 (2017): 87–98, 10.1007/978-1-4939-7030-8_8. 28500598

[58] K. A. Sauer , P. Scholtes , R. Karwot , and S. Finotto , “Isolation of CD4^+^ T Cells From Murine Lungs: A Method to Analyze Ongoing Immune Responses in the Lung,” Nature Protocols 1 (2006): 2870–2875, 10.1038/nprot.2006.435. 17406546

[59] F. Son , K. Umphred‐Wilson , J. H. Shim , and S. Adoro , “Assessment of ESCRT Protein CHMP5 Activity on Client Protein Ubiquitination by Immunoprecipitation and Western Blotting,” Methods in Molecular Biology 1998 (2019): 219–226, 10.1007/978-1-4939-9492-2_16. PMC10216709 31250305

