# Supplementary material for: CHMP5 Is a TCR‐ and Interleukin‐7‐Regulated Protein Required for Naïve T Cell Homeostasis
Source: Eur J Immunol. 2026 Nov 11;56(11):e70310. doi: 10.1002/eji.70310 (PMC13755503; doi:10.1002/eji.70310)
Supplement: Supplementary file 1 [file EJI-56-e70310-s001.pdf]

Figure S1: Characterization and immunophenotype of CHMP5-deficient T cells.
(A and **B)** Representative flow cytometry histograms **(A)** and quantification of percentages **(B)** of Cd8a-cre activity marked by GFP expression in thymic double negative (DN), DP, CD4 single-positive (SP), and CD8 SP (left), and splenic or lymph node $CD19^+$, $CD4^+$ T cells, or $CD8^+$ cells from *Cd8a*-Cre^+-$R26^{LSL\text{-}YFP}$ mice. **(C** and **D)** Representative flow cytometry graphs of the indicated surface proteins on $CD4^+TCR\beta^+$ and $CD8^+TCR\beta^+$ cells from spleen (**C**) and lymph nodes (**D**) of *Chmp5*^{Cd8a} mice. **(E** and **F)** Representative flow cytometry histograms **(E)** and quantification of percentages **(F)** of hCD2-cre activity marked by YFP expression in thymic DN, DP, CD4 SP, CD8 SP, and splenic and lymph node $CD19^+$, $CD4^+$ T cells, or $CD8^+$ cells isolated from hCD2-$iCre^+$-$R26^{LSL\text{-}YFP}$ mice. (**G** and **H**) Surface markers on $CD4^+$ and $CD8^+$ T cells from spleen (**G**) and lymph node (**H**) of *Chmp5*^{hCD2} mice. (**I**) Average (± SEM; *Chmp5*^{wt}, n = 6; *Chmp5*^{hCD2}, n = 4) frequency of T cells based on CD44 and CD62L expression. Two-way ANOVA with Tukey's multiple comparison: ns, not significant; **, $P < 0.01$; ****, $P < 0.0001$.

Figure S2: Assessment of apoptosis regulators and oxidative stress in experimental T cells.
(**A**-**E**) Geometric mean fluorescence intensity (MFI) of Bcl-2, Mcl-1, or Bax protein expression (**A** and **B**) or CellROX staining (**C**, **D**, and **E**) in the tdTomato versus GFP gated populations from $CD4^+$ and $CD8^+$ T cells from *R26*$^{mTmG/CreERT2}$*Chmp5*$^{fl/fl}$ or cre^- littermate control *R26*$^{mTmG/+}$*Chmp5*$^{fl/fl}$ mice 48 hours after *in vitro* tamoxifen treatment (**A** and **C**) or in $CD4^+$ and $CD8^+$ T cells isolated from spleens and lymph nodes of *Chmp5*^{hCD2} mice or *Chmp5*^{wt} littermate controls (**B** and **D**) or from spleen and thymus of bone marrow chimera mice reconstituted with B6.SJL (WT) and *Chmp5*^{wt} (WT) or *Chmp5*^{hCD2} (KO) bone marrow (**E**). ns, not significant; *, $P < 0.05$. Student's t-Test.

Supplementary Table S1: List of western blot antibodies

Supplementary Table S2: List of flow cytometry antibodies

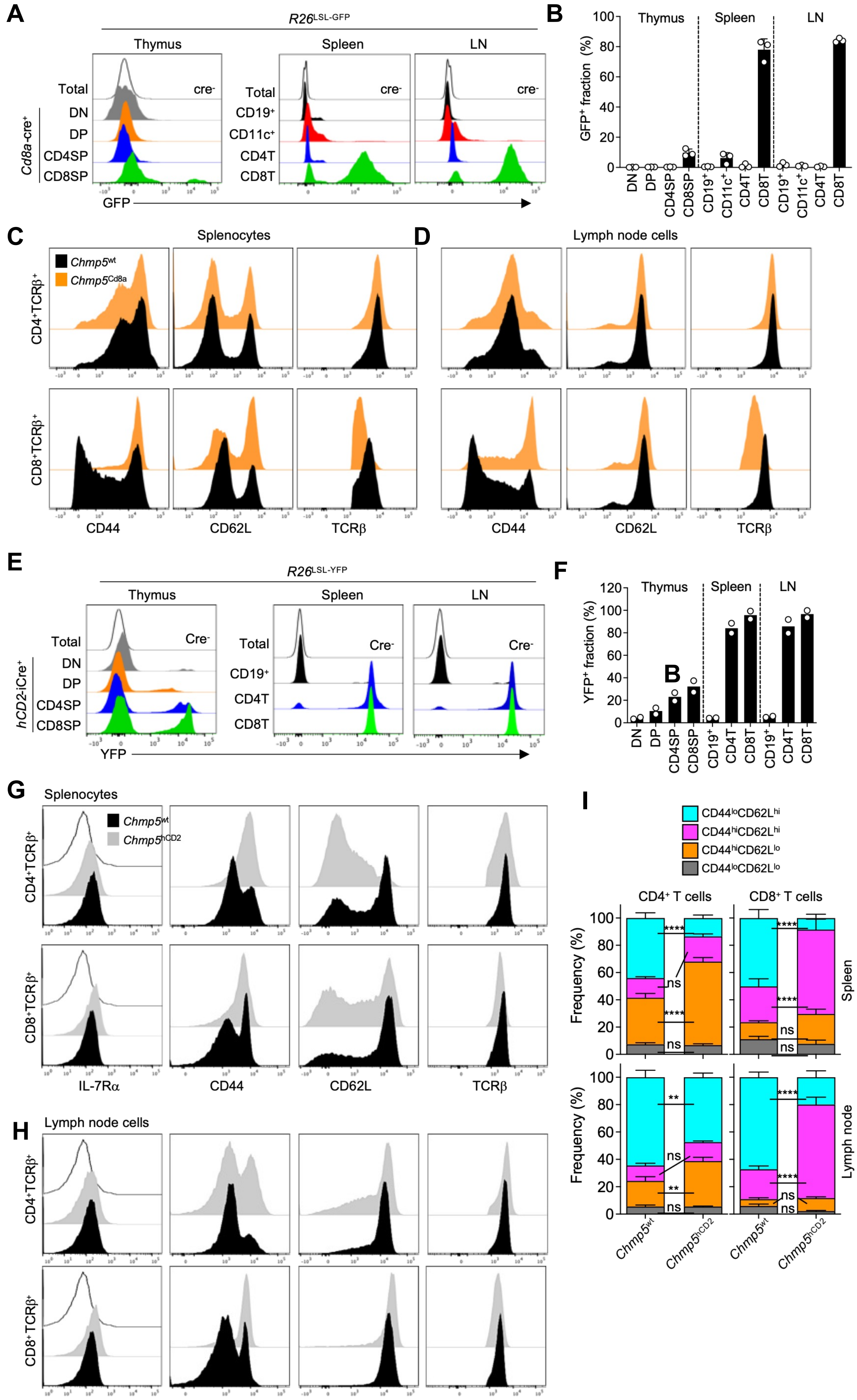

Figure S1: Characterization and immunophenotype of experimental CHMP5-deficient T cells.

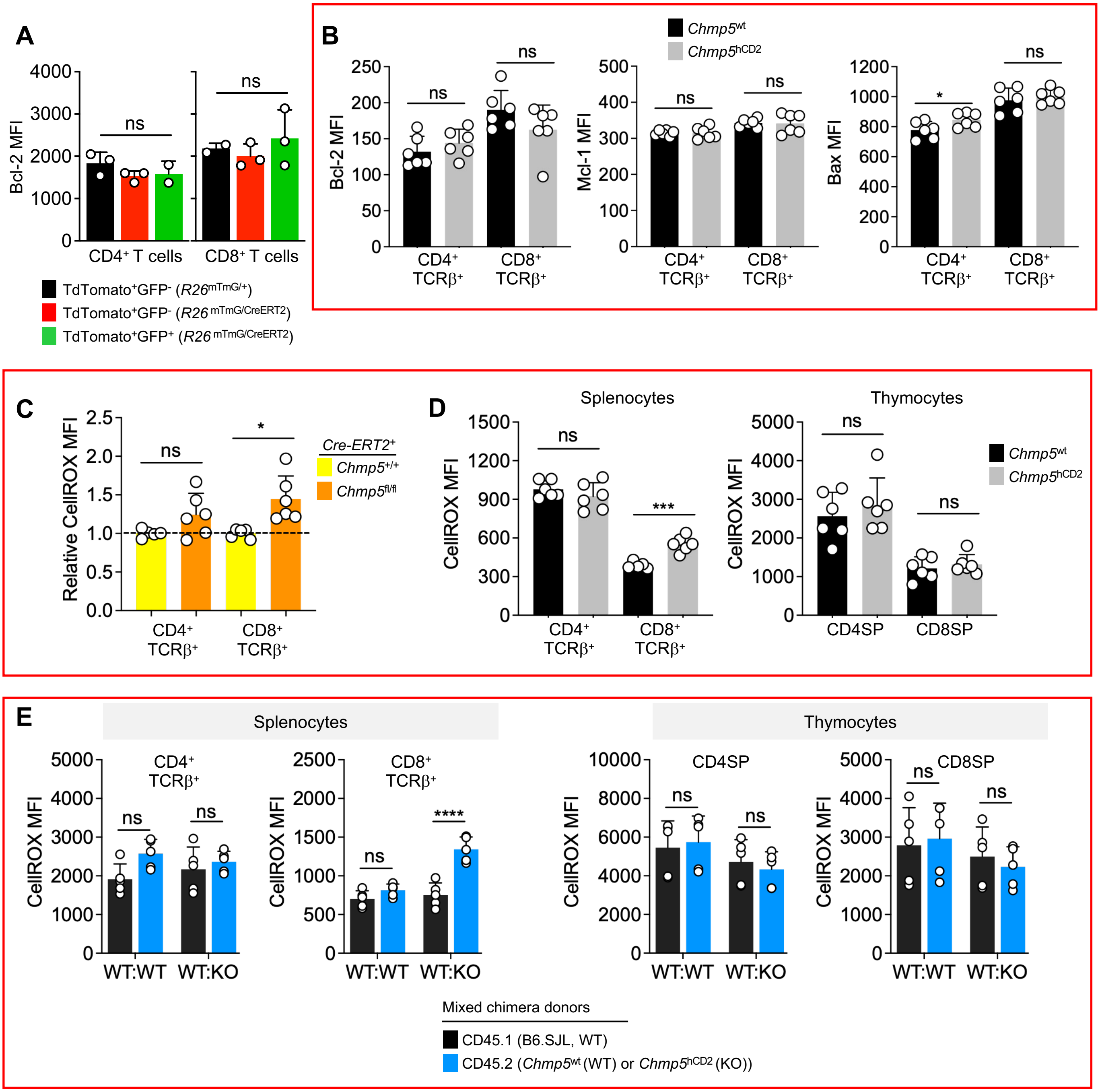

Figure S2: Assessment of apoptosis regulators and oxidative stress in experimental T cells.
